# The Neoclassical Growth Model and the Labor Share Decline

**DOI:** 10.1515/bejm-2020-0254

**Published:** 2021-04-12

**Authors:** Zachary L. Mahone, Joaquín Naval, Pau S. Pujolas

**Affiliations:** McMaster University, Hamilton, Canada; Universitat de Girona, Girona, Spain

**Keywords:** neoclassical growth model, labor share, model performance, E10, E13, E17, E20, E30

## Abstract

The labor share may be declining in the data, but it is often assumed constant in neoclassical growth models (NGM). We assess the quantitative importance of this discrepancy by comparing alternative calibration approaches featuring constant and declining labor shares. We find little difference in model performance. Our results derive from strong general equilibrium effects: while a declining labor share mechanically lowers wage growth, the investment response pushes wages back up. Hence, different models deliver nearly identical paths of macro aggregates. Numerous robustness checks (including a CES production function, different time periods, and calculations of the labor share) reinforce the similarity of performance across model specifications. We conclude that the NGM with a constant labor share is still an appropriate choice to study many standard macro aggregates.

## 1 Introduction

The neoclassical growth model (NGM), a workhorse of modern macroeconomics, typically features a Cobb–Douglas production function with a constant labor share.1This is almost verbatim the first sentence of Kehoe and Prescott (2007). As in Cole and Ohanian (2007), by the NGM “we mean the theory that Cass (1965) and Koopmans (1965) set out, augmented with […] shocks […] as in Kydland and Prescott (1982),” as (p. 21). Recently however, a stream of literature has argued that labor’s share of output is steadily declining in the data. In this paper we ask to what extent the NGM’s performance is sensitive to this decline. Our main finding is that the performance of the NGM with a constant labor share is similar to versions that explicitly include time-varying labor shares. Our result is robust to using a CES production function, which is the technology of choice in much of the labor share literature. Moreover, the result is also robust to focusing on different time periods, or alternative manners of computing the labor share. The conclusion of our analysis is that for studying the paths of standard macro aggregates and prices the NGM with a constant labor share is still an appropriate choice.

We measure performance as the relative errors between model and data across a standard set of macro aggregates, including quantities (*C*_*t*_, *I*_*t*_, *K*_*t*_, *L*_*t*_), prices (*r*_*t*_, *w*_*t*_) and output per worker (*Y*_*t*_/*L*_*t*_). We then simulate three versions of the model (feeding in a constant labor share, the exact evolution of the labor share, and a trend decline) and find that performance of the NGM is largely unaffected. Importantly, feeding in the empirical evolution of the labor share does not uniformly improve model performance. Moreover, our results highlight the importance of general equilibrium effects. For example, our exercise reveals an interesting link between the labor share and equilibrium wage paths. Although one might expect a declining labor share to reproduce a degree of wage stagnation, as in the data, forward looking agents perceive an increased rate of return and accumulate additional capital, raising labor’s marginal product and wages. The result is that the NGM with a declining labor share yields a time path of wages nearly identical to the NGM with a constant labor share, both predicting excessive wage growth. Our results then are twofold. First, the constant labor share assumption of the NGM is fairly innocuous, at least for paths of macro aggregates. Second, this is driven by strong equilibrium responses to the labor share decline.

We view our results as complementing the existing literature. While most of the literature tries to explain the labor share decline, we take the decline as given to instead focus on its implications for other macro aggregates through the lens of a benchmark model. The message of our quantitative exercises is that incorporating the labor share decline has little impact on these aggregates. Our finding is particularly relevant given the large number of quantitative macro models with the NGM at their foundation.

The well-known ingredients of the NGM include a rational, forward-looking, representative household that decides how much to work, invest and consume; a representative firm that produces a final good operating a production function with capital and labor; and perfectly competitive markets. In addition, it has become standard practice to assume, as we will, that producers operate a Cobb–Douglas technology with a constant capital share parameter *α*, appealing to what Kaldor (1957) noted as the empirical labor share’s “remarkable constancy in ‘developed’ economies” (p. 591). With a declining labor share however, one might argue that this is less a feature and more a bug. The goal of this paper is to quantify the cost of this bug and, more generally, to evaluate the relevance of the labor share decline to the broader macro literature through the lens of a widely used model.

In the NGM laid out above, the labor share is governed by parameter *α* appearing in the exponents of the Cobb–Douglas production function.2The assumption of a competitive labor market makes the wage equal the marginal product of labor, *w* = ∂*Y*/∂*L*. A Cobb–Douglas production function, *Y* = *AK* ^*α*^ *L* ^1−*α*^, makes the marginal product of labor be ∂*Y*/∂*L* = (1 − *α*)*Y*/*L*. Combining them both, we get that the labor share, *wL*/*Y*, is constant and equal to 1 − *α*. Since this is typically assumed constant across periods, the model’s labor share does not decline. We confront the NGM with a declining labor share by using a vector $\alpha =\left(\alpha_{1},\alpha_{2},\ldots ,\alpha_{T}\right)$ that allows us to replicate its evolution. Because *α* governs how capital and labor combine to produce output, each vector *α* requires a separately calibrated vector of TFP *A* = (*A*_1_, *A*_2_, …, *A*_*T*_) to be internally consistent with measured output. Beyond these pairs (*α*, *A*) however, no further adjustments are made across calibrations and none of the macro variables used to assess performance are pinned down through targets.

Using the pairs (*α*, *A*), we perform a series of simulation exercises and ask how model performance is affected across a variety of macro variables predicted by the model (wage, rental rate, investment, consumption, capital, hours worked, and output per worker). We find that differences in performance, measured as relative errors between model and data series, are negligible across alternative versions of the model. For many variables the constant labor share actually performs marginally better, while consumption, rental rates and wages appear to be the most sensitive, and most improved, by feeding in the exact evolution of the labor share. In particular, our findings suggest that studies focused on the evolution of hours worked would most benefit from accounting for the labor share decline, while the constant labor share is preferred for capital and investment dynamics. To further investigate our main results, we decompose the macro variables into trend and cyclical components and ask whether either is the primary driver of our findings. We find that our main results are in fact echoed for both the trend and cycle – the NGM with a fixed labor share is a good choice in either case. It should be emphasized that these results do not suggest that the NGM is insensitive to values of *α* generally, but rather that in the context of a calibrated model the size of the measured decline in the labor share simply has little impact.

We perform several robustness checks to further assess our main results. First and foremost, the Great Recession period seems to dramatically affect most variables negatively. When we remove that period from our analysis, the results stay the same. Second, we consider an alternative approach to computing the labor share, but this does not alter the findings either. Last but not least, we allow for a CES production technology and falling price of investment. Repeating our benchmark exercise, the results are again unchanged.

While the issue of the LS decline has received much attention, it is itself a point of contention in the literature. Koh, Santaeulàlia-Llopis, and Zheng (2020, henceforth KSZ) use a revised version of the national income and product accounts (NIPA) to argue that the observed empirical decline in the labor share can be entirely explained by the capitalization of intellectual property products. Our paper provides initial model-based evidence that, even if the labor share were declining, it would not appreciably impact the paths of macro aggregates predicted by the NGM.

Karabarbounis and Neiman (2014, henceforth KN) are among the first to present empirical evidence of a decline and argue that half of the decline in the labor share can be accounted for by a decrease in the price of investment goods together with an elasticity of capital to labor larger than one. Since then, a number of alternative hypotheses have been proposed to explain this decline. Elsby, Hobijn, and Sahin (2013) claim that a third of the decline appears to be an artefact of mismeasurement and propose the offshoring of labor-intensive components in the U.S. supply chain as their preferred hypothesis to explain the decline. Glover and Short (2018) emphasize that the demographic composition of the labor force can account for roughly half of the decline in the labor share. Autor et al. (2017) propose that a few, low-labor share, large market share, superstar firms are responsible for the decline. Finally, Grossman et al. (2017) explore the possibility that the global productivity slowdown has directly led to a fall in the labor share. Rather than advancing an economic theory of the labor share decline, in our paper we quantify its relevance to other macro variables through the lens of a benchmark model. To do this, we introduce a declining labor share in the most straightforward way the NGM permits. Our results are complementary to the existing literature, providing context through which we better understand the macroeconomic implications of a falling labor share.

In Section 2 we explain the model and data used in the paper, which follow the methodology in Conesa, Kehoe, and Ruhl (2007, henceforth referred to as CKR). In Section 3 we show our benchmark results for the United States since the post-war period. In Section 4 we perform the robustness exercise. In Section 5 we highlight the general equilibrium effects crucial for our results by focusing on wage paths. Section 6 concludes.

## 2 Model and Data

In this section we first define the NGM, then briefly discuss how we construct the data series and calibrate the model. All steps are in line with CKR.

### 2.1 The Neoclassical Growth Model

The version of the NGM we use in this paper consists of a representative household, endowed with initial capital $K_{t_{0}}$ , hours per year, $\bar{h}$ , and total working age population, *N*_*t*_, that takes the prices of labor, *w*_*t*_, and rental rate of capital, *r*_*t*_, as given, and chooses how much to consume, *C*_*t*_, work, *L*_*t*_, and invest (in the form of next period’s capital), *K*_*t*+1_, to maximize her discounted flow of utility,

(1) $$\max_{{\left\{C_{t},L_{t},K_{t+1}\right\}}_{t=t_{0}}^{\infty }}\overset{\infty }{\underset{t=t_{0}}{\sum }}\beta^{t}\left(\gamma \;\log\left(C_{t}\right)+\left(1-\gamma \right)\log\left(\bar{h}N_{t}-L_{t}\right)\right)$$

such that ∀ *t* ≥ *t*_0_

(2) $$C_{t}+K_{t+1}\leq w_{t}L_{t}+\left(1-\delta +r_{t}\right)K_{t},$$

(3) $$C_{t},K_{t+1},L_{t}\geq 0,L_{t}\leq \bar{h}N_{t},$$

where *γ* governs the relative weight of consumption over leisure, *β* is the discount factor, and *δ* is the depreciation rate of capital. There is a perfectly competitive, representative firm that hires labor and rents capital to operate a Cobb–Douglas production function, $Y_{t}=A_{t}K_{t}^{\alpha_{t}}L_{t}^{1-\alpha_{t}}$ , where *A*_*t*_ is an exogenous productivity, implying that the wage and rental rate are given by

(4) $$w_{t}=\left(1-\alpha_{t}\right)A_{t}K_{t}^{\alpha_{t}}L_{t}^{-\alpha_{t}},$$

(5) $$r_{t}=\alpha_{t}A_{t}K_{t}^{\alpha_{t}-1}L_{t}^{1-\alpha_{t}}.$$

Last, feasibility is satisfied,

(6) $$C_{t}+K_{t+1}=Y_{t}+\left(1-\delta \right)K_{t}.$$

Next, we show how to map this model to the data, in order to evaluate different versions of the model.

### 2.2 Data

Our benchmark analysis is 1955–2018. We start in 1955 because of employment data limitations, and finish in 2018 because that is the last year of available data we have. The empirical macroeconomic variables that we use are the interest rate, the wage, investment, consumption, capital and hours worked. Using superscript *d* to denote data values, these are ${\left\{L_{t}^{d},I_{t}^{d},C_{t}^{d},K_{t}^{d},r_{t}^{d},w_{t}^{d}\right\}}_{t=1955}^{2018}$ . We obtain our data from the Bureau of Economic Analysis, the United Nations, and Stats OECD datasets.

Empirical hours worked, $L_{t}^{d}$ , is computed by multiplying the number of workers by average hours worked.

We next compute the empirical series for investment, $I_{t}^{d}$ , and consumption, $C_{t}^{d}$ . Both variables, taken directly from the national accounts, are expressed in current prices and deflated using the GDP deflator. To construct the latter, we take GDP from the national accounts in both constant prices, $Y_{t}^{d,c}$ , and nominal prices, $Y_{t}^{d,n}$ . Investment is constructed using gross capital formation, ${\text{GCF}}_{t}^{d}$ , according to $I_{t}^{d}={\text{GCF}}_{t}^{d}\times Y_{t}^{d,c}/Y_{t}^{d,n}$ . Since the model is a closed economy with no government, consumption is computed as $C_{t}^{d}=Y_{t}^{d,c}-I_{t}^{d}$ .

We next turn to capital, $K_{t}^{d}$ . The perpetual inventory method yields the law of motion

(7) $$K_{t+1}^{d}=\left(1-\delta \right)K_{t}^{d}+I_{t}^{d},$$

that requires the series $I_{t}^{d}$ (computed above), the depreciation rate *δ* (computed in the calibration section below), and initial capital $K_{t_{0}}=K_{1955}^{d}$ . As the model is sensitive to the initial capital level, we compute the capital stock by imposing a capital-output ratio in 1955 equal to the average over the years 1956–1965. Namely,

(8) $$\frac{K_{1955}^{d}}{Y_{1955}^{d,c}}=\frac{1}{10}\overset{1965}{\underset{t=1956}{\sum }}\frac{K_{t}^{d}}{Y_{t}^{d,c}},$$

which yields $K_{1955}^{d}=2.76Y_{1955}^{d,c}$ .

Finally, we compute the series for prices. Consistent with the model, the wage is given by

(9) $$w_{t}^{d}=\frac{\left(1-\alpha_{t}^{d}\right)Y_{t}^{d,c}}{L_{t}^{d}},$$

and the interest rate by

(10) $$r_{t}^{d}=\frac{\alpha_{t}^{d}Y_{t}^{d,c}}{K_{t}^{d}}.$$

To compute these series we are missing only the series for $\alpha_{t}^{d}$ ; in the benchmark, we use the labor share data provided by Koh et al. (2020).3In Section 4 we perform a robustness check computing the labor share in line with CKR.


### 2.3 Calibration

To simulate model counterparts to the six series computed above, the NGM requires parameters *β* (the discount factor), *γ* (the consumption-leisure weight), and *δ* (the depreciation rate); initial condition *K*_1955_; balanced growth path parameters *g*_*N*_ (long-run population growth) and *g*_*A*_ (long-run TFP growth); and the series ${\left\{\alpha_{t}\right\}}_{t=1955}^{2018}$ (capital shares), ${\left\{N_{t}\right\}}_{t=1955}^{2018}$ (population), and ${\left\{A_{t}\right\}}_{t=1955}^{2018}$ (TFP). It is important to stress that the only variables whose values depend on the series ${\left\{\alpha_{t}\right\}}_{t=1955}^{2018}$ are ${\left\{A_{t}\right\}}_{t=1955}^{2018}$ and *g*_*A*_. We now describe how we perform the calibration, saving these last two for the end.

Note that the Euler equation and the labor supply equation that arise from maximizing Eq. (1) subject to Eq. (2) are given by

(11) $$\frac{1}{C_{t}}=\beta \left(1-\delta +r_{t+1}\right)\times \frac{1}{C_{t+1}},$$

and

(12) $$\frac{\gamma }{C_{t}}=\frac{1-\gamma }{w_{t}\left(\bar{h}N_{t}-L_{t}\right)}.$$

We use the first expression to solve for *β*, the second one to solve for *γ*, and then calibrate the two parameters by averaging for the period 1955–1964. Namely,

(13) $$\beta =\frac{1}{10}\overset{1964}{\underset{t=1955}{\sum }}\frac{1}{1-\delta +r_{t+1}^{d}}\times \frac{C_{t+1}^{d}}{C_{t}^{d}}=0.9715,$$

and

(14) $$\gamma =\frac{1}{10}\overset{1964}{\underset{t=1955}{\sum }}\frac{C_{t}^{d}}{w_{t}^{d}\left(\bar{h}N_{t}^{d}-L_{t}^{d}\right)+C_{t}^{d}}=0.2984.$$

To compute *δ*, we use the empirical series of consumption of fixed capital, ${\text{CFC}}_{t}^{d}$ , and the series of GDP and capital computed in the previous section. We set *δ* so that, on average, it depreciates a fraction of GDP consistent with the data. Namely,

(15) $$\frac{1}{10}\overset{1964}{\underset{t=1955}{\sum }}\frac{{\text{CFC}}_{t}^{d}}{Y_{t}^{d,n}}=\frac{1}{10}\overset{1964}{\underset{t=1955}{\sum }}\frac{\delta K_{t}^{d}}{Y_{t}^{d,c}}.$$

This yields *δ* = 0.0516. The initial condition *K*_1955_ comes directly from the series $K_{t}^{d}$ .

The sequence of working age population, $N_{t}^{d}$ , is taken directly from the data, and its long-run growth rate, $g_{N}^{d}=0.0041$ is the growth rate of the last year, 2017 to 2018.

The calibration strategy thus far depends only on empirical series. Hence, the values computed above will be used across all versions of the NGM. However, calibration of the remaining parameters, ${\left\{\alpha_{t}\right\}}_{t=1955}^{2018}$ , ${\left\{A_{t}\right\}}_{t=1955}^{2018}$ and *g*_*A*_, depend on the particular version being considered.

We analyze three different versions of the NGM. In the first, *Constant*, the labor share is constant and equal to its average during the period 1955–2018. Namely,

(16) $$1-\alpha^{cons}=1-\frac{1}{64}\overset{2018}{\underset{t=1955}{\sum }}\alpha_{t}^{d}=0.6471.$$

The second, *Trend*, features a labor share that linearly connects the first and last terms of the $1-\alpha_{t}^{d}$ series. Specifically,

(17) $$1-\alpha_{t}^{trend}=1-\left(\alpha_{1955}^{d}+\frac{\alpha_{2018}^{d}-\alpha_{1955}^{d}}{64}t\right)=0.6590-0.0006127\left(t-1955\right).$$

**Figure 1:**
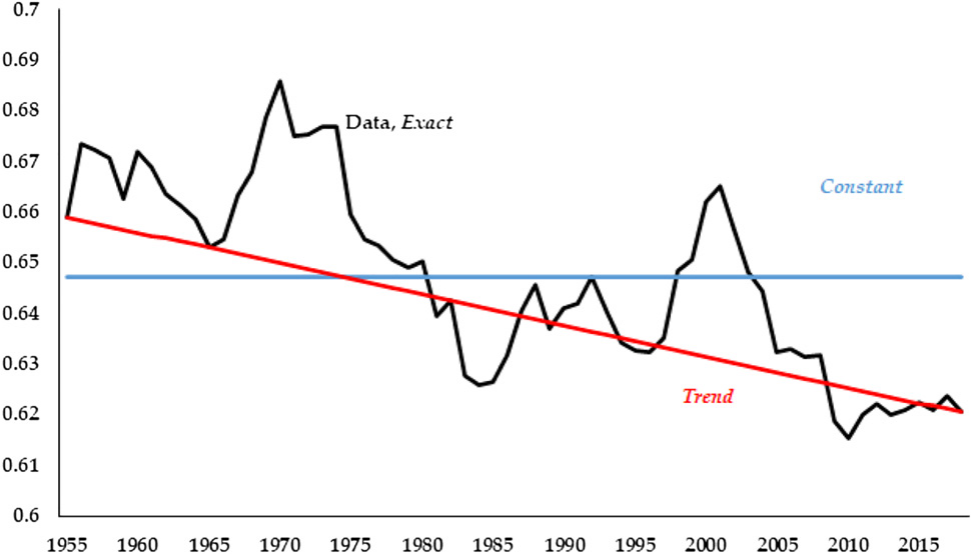
Different se-ries for the labor share.

Finally, the third, *Exact*, feeds in the labor share from the data, Eq. (21). We plot the three series of the labor share in Figure 1.

TFP is computed as a residual that depends on the series of capital, labor, GDP, and calibrated *α*. Namely, $A_{t}^{i}$ , is a function of the calibrated $\alpha_{t}^{i}$ , *i* ∈ {*cons*, *trend*, *exact*}, and is given by

(18) $$A_{t}^{i}=\frac{Y_{t}^{d,c}}{{\left(K_{t}^{d}\right)}^{\alpha_{t}^{i}}{\left(L_{t}^{d}\right)}^{1-\alpha_{t}^{i}}}.$$

Computing the model requires a growth rate of TFP after year 2018, which we refer to as $g_{A}^{i}$ , and set equal to the average growth rate of $A_{t}^{i}$ during the period 1955–2018.4The values of this growth rate are $g_{A}^{cons}=0.01093$ , $g_{A}^{trend}=0.01047$ , and $g_{A}^{exact}=0.01047$ . Each exercise uses $\alpha_{t}^{i}$ , $A_{t}^{i}$ and $g_{A}^{i}$ in simulating the model version *i*.

It is important to stress that we cannot separately calibrate a TFP series from the series of *α*. To see why, suppose that *A* ^*i*^ was the same in all the versions (and, for the sake of argument, it was the one derived from having a constant parameter, *α* ^*cons*^, making it *A* ^*cons*^). Further, suppose there was a different version of the model, version *i*, that perfectly predicted the paths of capital, $K_{t}^{i}=K_{t}^{d}$ , and labor, $L_{t}^{i}=L_{t}^{d}$ , while having a different path for *α*, *α* ^*i*^ ≠ *α* ^*cons*^. Then, this version *i* would wrongly predict aggregate output,

$$\begin{array}{cc} Y_{t}^{i} & =A_{t}^{cons}{\left(K_{t}^{d}\right)}^{\alpha_{t}^{i}}{\left(L_{t}^{d}\right)}^{1-\alpha_{t}^{i}}=\frac{Y_{t}^{d,c}}{{\left(K_{t}^{d}\right)}^{\alpha_{t}^{C}}{\left(L_{t}^{d}\right)}^{1-\alpha_{t}^{C}}}{\left(K_{t}^{d}\right)}^{\alpha_{t}^{i}}{\left(L_{t}^{d}\right)}^{1-\alpha_{t}^{i}}\\ & =Y_{t}^{d,c}{\left(K_{t}^{d}\right)}^{\alpha_{t}^{i}-\alpha_{t}^{cons}}{\left(L_{t}^{d}\right)}^{\alpha_{t}^{cons}-\alpha_{t}^{i}}\neq Y_{t}^{d,c}. \end{array}$$

Using a similar argument, the predicted paths for wages, *w*_*t*_, and rental rates, *r*_*t*_, would be off. Moreover, a wrong prediction of aggregate output also implies that either the series of consumption or the series of investment (or both) are wrong, because $Y_{t}^{i}=C_{t}^{i}+I_{t}^{i}$ .

Last, we standardize all the series so that in 1955 *K*_1955_ = 1 and *L*_1955_ = 1. We do this normalization, similar to Santaeulàlia and Rios-Rull (2010), because when the coefficients in a Cobb–Douglas production function are allowed to change over time, as we do in our exercise, the units of the capital–labor ratio affect the results. By normalizing them to one in the first period, the impact of this problem is minimal.

## 3 Model Comparison

In this section we compare the performance of different versions of the NGM featuring alternative paths of the labor share parameter *α*. Performance is measured as the relative error from the data,

(19) $$\sigma^{i}\left(x\right)=\frac{1}{64}\sqrt{\overset{2018}{\underset{t=1955}{\sum }}{\left(\frac{x_{t}^{i}-x_{t}^{d}}{x_{t}^{d}}\right)}^{2}},$$

where *x* stands for wage *w*, interest rate *r*, consumption *C*, investment *I*, capital *K*, hours worked *L*, and labor productivity *Y*/*L*; ${\left\{x_{t}^{d}\right\}}_{t=1955}^{2018}$ is the variable’s empirical counterpart; and *i* ∈ {*cons*, *trend*, *exact*}.5To compute the numerical solution of the model, we modify the Matlab codes developed by Kim Ruhl for CKR. We use the last version available, from April 2008, which can be freely downloaded from greatdepressionsbook.com. Better performance implies smaller values and closer predictions to the data. Our results are robust to using absolute distances rather than euclidean distances in the relative error measure.

Our main results are in Table 1, which reports the relative errors for each simulation-variable pair. Rows reflect the three different versions of the NGM and columns report the six different macro aggregates analyzed. For each macro aggregate, the model version that performs best is highlighted in bold. A quick look at the bold values shows that no single version of the NGM dominates.

**Table 1:** Relative errors, smallest values in bold.

	*r*	*w*	*I*	*C*	*K*	*L*	*Y*/*L*
Constant	0.1631	0.1037	**0.5378**	0.1016	**0.4335**	**0.1834**	**0.0874**
Trend	0.1580	0.1071	0.6311	0.0978	0.5021	0.1877	0.1113
Exact	**0.1540**	**0.1035**	0.6090	**0.0977**	0.4701	0.1937	0.1035

Further examination of Table 1 shows that *Constant* performs best for investment, capital, hours worked and labor productivity, whereas *Exact* dominates in interest rate, wage, and consumption; by contrast, *Trend* does not dominate for any variable. However, it comes second with wage, interest rate, consumption, and hours worked.

**Figure 2:**
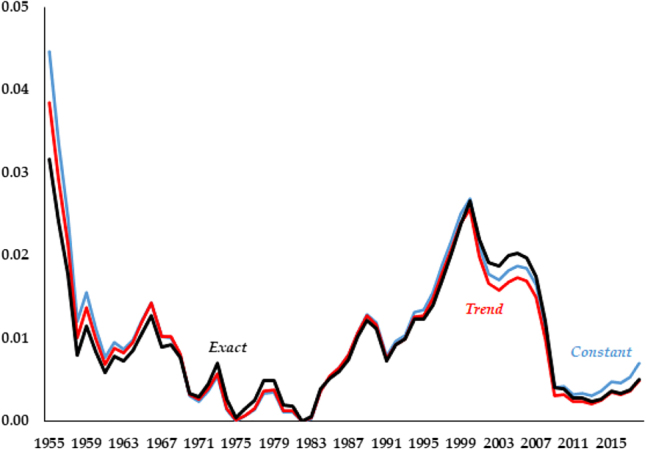
Consumption relative errors.

This lack of dominance of one model versus another can be further emphasized by the fact that the variation in performance across models is rather small. As an example, in Figure 2 we plot the series of simulated differences in consumption paths for each model against the data. While Table 1 shows *Exact* performs best, the differences are very small, quantitatively and visually. The three simulated paths for consumption lie nearly on top of each other. In fact, the series for *Exact* dominates the beginning of the series up until 1970, but for all the other time periods it performs either worse (late 1970s, early 2000s) or indistinguishably equal to the second best.

**Figure 3:**
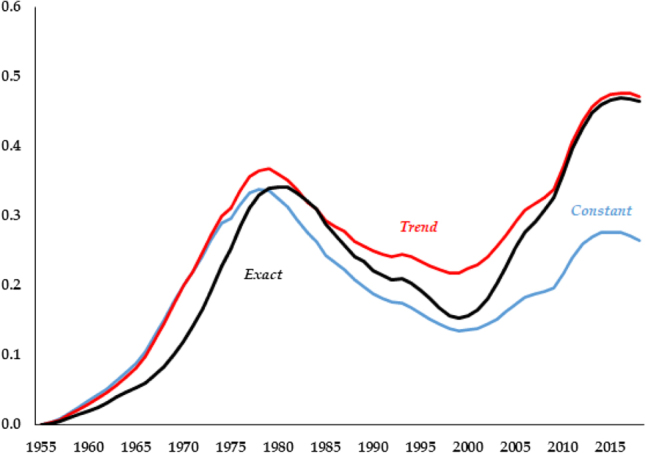
Capital relative errors.

The largest relative errors across models arise in the capital series. As is clear in Figure 3, the series with a constant labor share yields smallest relative errors from the 1980s onwards. That being said, it is the series that fares worst at the beginning of the period, with the *Exact* faring much better.

**Figure 4:**
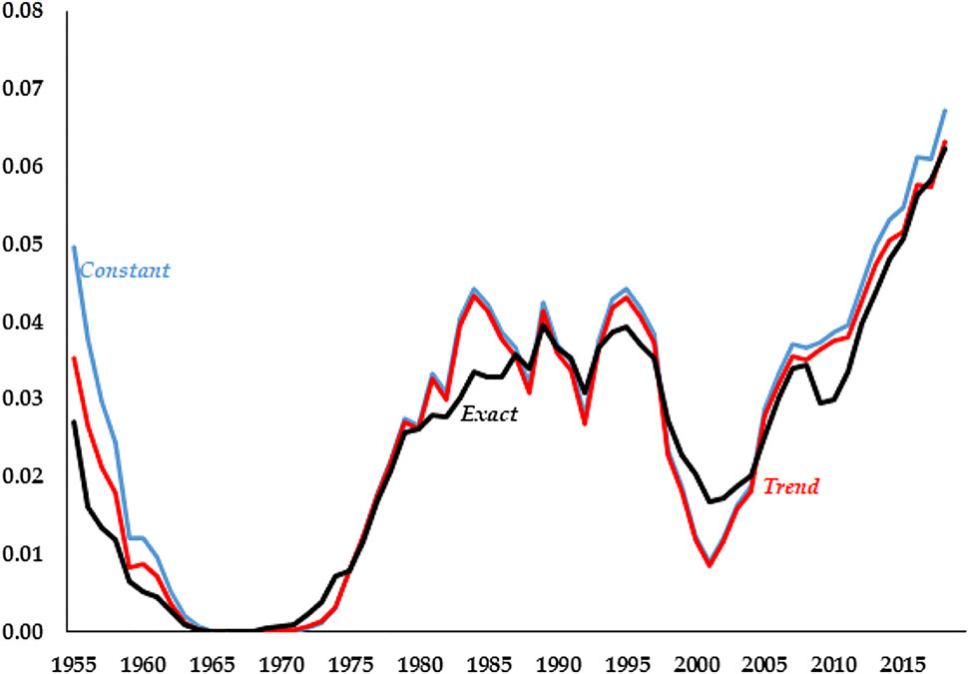
Interest rate relative errors.

**Figure 5:**
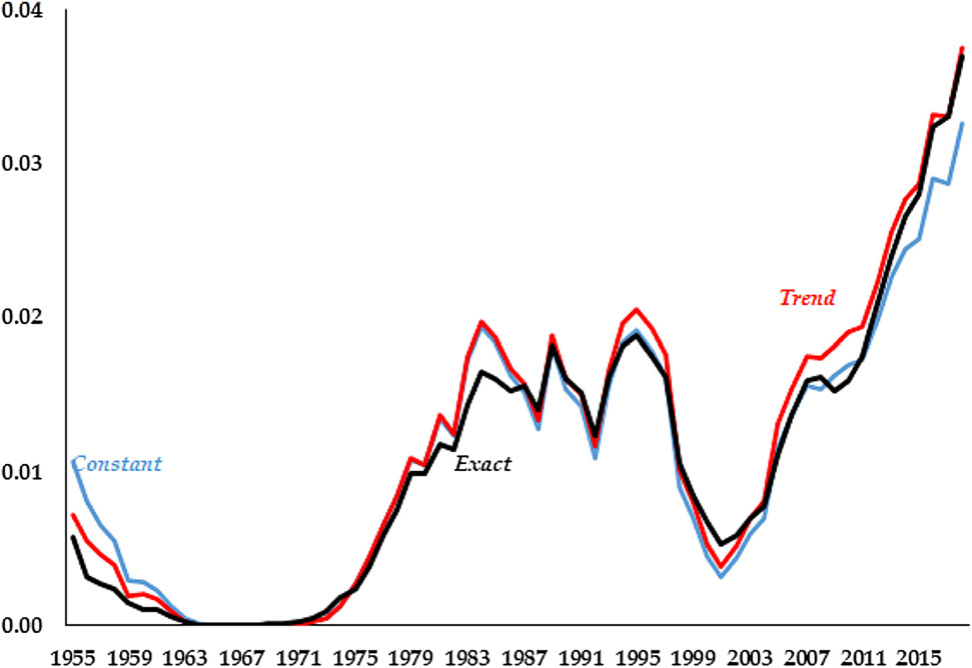
Wage relative errors.

The analysis for the interest rate, Figure 4, and the wage, Figure 5, are very similar. In both cases *Exact* performs best. But again, the series of errors across models is very similar, with the series crossing multiple times. Notably, the difference between model and data is largest after 2007 across all models, with the errors seemingly exploding at the end of the sample.

The main results of Table 1 convey a clear message. Incorporating the labor share decline into the NGM has minimal impact on the paths of macro aggregates studied. The errors that each model makes when compared to the original data are highly correlated.

### 3.1 Trend and Cycle Components

So far our analysis has studied how the overall path of macro aggregates compares between model and data, and we have seen that the model with a constant labor share remains a good choice. However, because many questions of interest distinguish between trend and cycle, it is useful to ask to what extent these results carry over to separate analysis of the cyclical and trend components of the macro aggregates we consider. In order to not generate confusion with the exercise *Trend*, we refer to the two objects in this exercise as the trend component (TC) and the cycle component (CC).

We perform a decomposition for each series *x*, defining the TC as

$${\text{TC}}_{x}=\left(x_{2018}-x_{1955}\right)/63,$$

and CC as

$${\text{CC}}_{x,t}=x_{t}-\left(x_{1955}+{\text{TC}}_{x}*\left(t-1955\right)\right).$$

We then produce the same model comparisons as above for each component separately.

Table 2 reports the results of this comparison for the TC components, where errors are computed as absolute errors. We do not compute relative errors as in Table 1 to avoid division by zero.

**Table 2:** Absolute errors of trend component.

	*r*	*w*	*I*	*C*	*K*	*L*	*Y*/*L*
Constant	0.0010	**0.0023**	**0.0021**	**0.0047**	**0.0544**	0.0023	**0.0027**
Trend	0.0009	0.0023	0.0037	0.0051	0.0725	0.0027	0.0037
Exact	**0.0009**	0.0023	0.0039	0.0049	0.0720	**0.0021**	0.0037

As before, we use bold numbers for the smallest errors. The pattern that emerges is very similar to the ones from Table 1, where no model dominates in all the categories but the model with a constant labor share has the smallest errors for wage (at six decimal points), investment, consumption, capital, and output per worker. The model using the exact labor share has the smallest error for both hours worked and the interest rate (at five decimal points). Moreover, Table 2 finds relatively small variation in performance across models for the TC, consistent with Table 1.

Turning to the CC, Table 3 reports the results of our analysis where errors are again computed as the sum of squared errors.6This is equivalent to absolute errors in the case of scalars such as the TC.


**Table 3:** Sum of squared errors of cycle component.

	*r*	*w*	*I*	*C*	*K*	*L*	*Y*/*L*
Constant	0.0145	**0.0160**	**0.0481**	**0.0448**	**0.4078**	**0.0828**	**0.0186**
Trend	0.0128	0.0182	0.0547	0.0504	0.6706	0.0843	0.0224
Exact	**0.0115**	0.0185	0.0648	0.0532	0.7782	0.1076	0.0316

The results are again very similar, where the model with a constant labor share has the smallest errors for wage, investment, consumption, capital, output per worker and also hours worked. By contrast, the model with the exact labor share has the smallest errors for the interest rate.

Combining this analysis with our main results, we find that the NGM with a constant labor share is still a good choice of model when exploring the behavior of macro aggregates. Moreover, this remains true whether one is interested in cylical or slope dynamics. However, our results may be affected by multiple objects. First, given the performance of wage and interest rate, one might be suspicious about the period of and after the Great Recession (all models do poorly then). Second, we use the labor share series from Koh et al. (2020), but there are alternative ways to compute the labor share, in particular we can follow CKR. Third, we can use a different production function, with the CES being the most widely-used in the literature. In the following section we treat each of these concerns in turn. We find our results are robust to all of them.

## 4 Robustness Analysis

### 4.1 Removing the Great Recession Period

**Figure 6:**
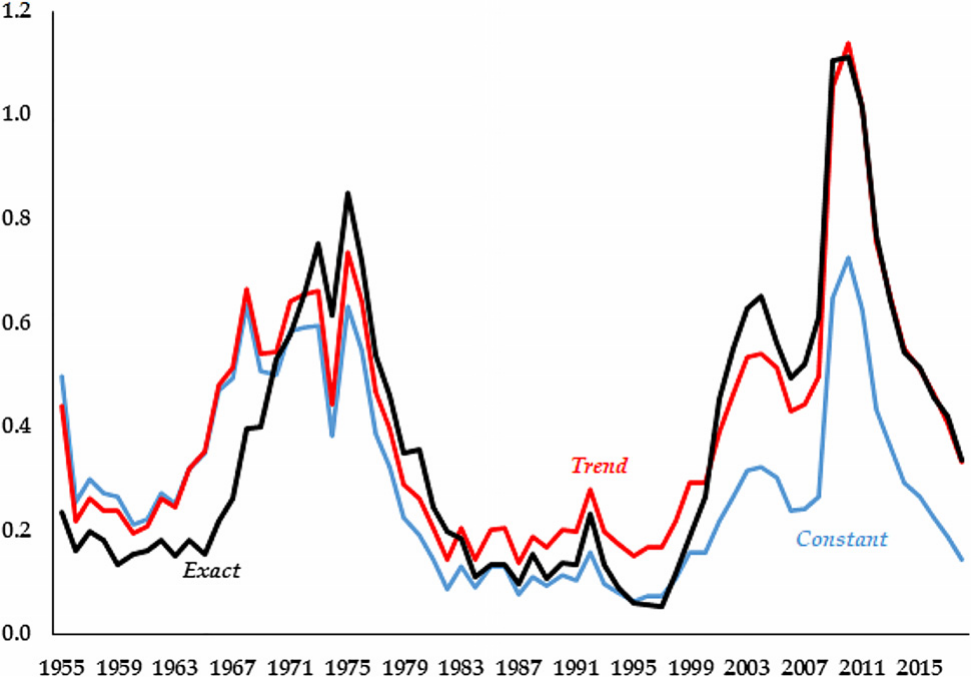
Investment relative errors.

**Figure 7:**
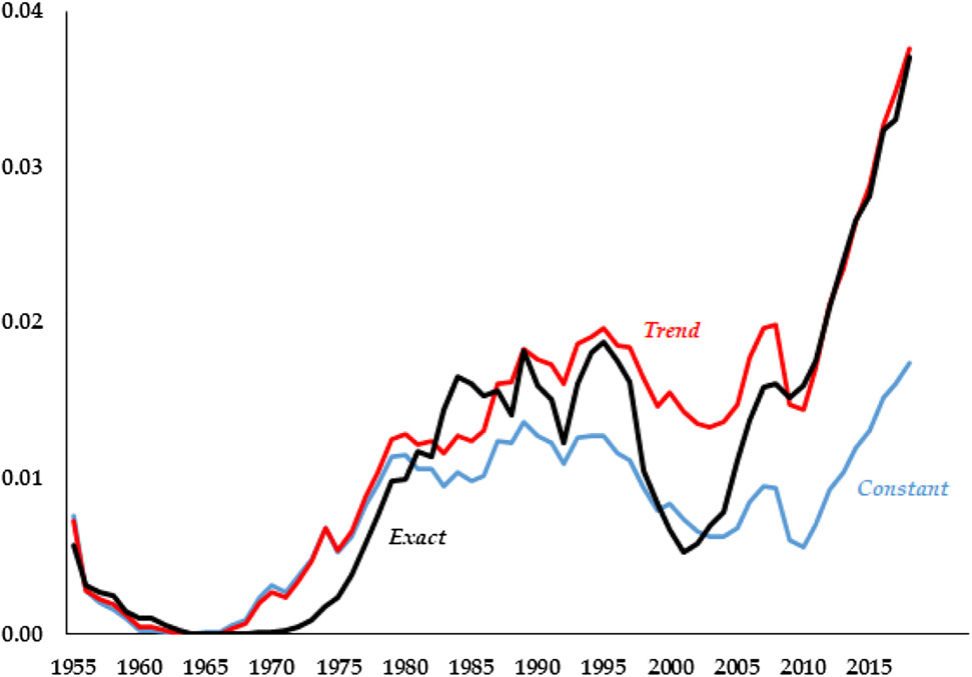
Output per worker relative errors.

The Great Recession occurred in the last years of our analysis and may be biasing our results. For example, in the capital series of Figure 3, while none of the models does particularly well after the mid-2000s, *Constant* deviates approximately half of what *Trend* and *Exact* do. While the consumption series of Figure 2 does better in that period than right before it, this is not the case for investment, see Figure 6. As we mentioned above, all the models do poorly for wages and interest rates in that period. Similarly, output per worker does particularly poorly during that period, most notably *Trend* and *Exact*, as can be seen in Figure 7.

These results may be more a statement about the extraordinary time period from 2007 onwards than they are a favorable result for the dominant model version. To check whether our results are driven by this, Table 4 reproduces Table 1 for the period 1955–2006 only. The same results emerge: no single model version dominates and the differences in performance are minimal. In fact, the results are qualitatively the same: *Constant* outperforms the other two for Investment, capital, hours worked and output per worker, whereas *Exact* outperforms the other ones for the wage, the interest rate, and consumption. As before, *Trend* comes in second place for a few variables.

**Table 4:** Relative errors, no Great Recession.

	*r*	*w*	*I*	*C*	*K*	*L*	*Y*/*L*
Constant	0.1469	0.0903	**0.5207**	0.1066	**0.4183**	**0.1925**	**0.0830**
Trend	0.1417	0.0914	0.5832	0.1034	0.4623	0.1984	0.0982
Exact	**0.1385**	**0.0879**	0.5494	**0.1025**	0.4220	0.2054	0.0879

### 4.2 CKR Labor Share

In all our analysis so far we have used the labor share from Koh et al. (2020). There are two good reasons for doing this. The first one is that they carefully computed the labor share consistently. The second is that their data allow us to perform our analysis beginning in 1955. Our model and calibration approach, however, have followed CKR. A natural question to ask, then is whether the results are driven by this differential treatment of the labor share versus the other parameters. A note of caution with this robustness exercise however, is that in following CKR, our analysis must begin in 1970 because of data availability.

CKR note that if the data were perfectly consistent with the model outlined above, we would compute period *t*’s labor share as the fraction of GDP used for compensation of employees, ${\text{CE}}_{t}^{d}$ . Namely,

(20) $$1-\alpha_{t}^{d}=\frac{{\text{CE}}_{t}^{d}}{Y_{t}^{d,n}}.$$

However, GDP at market prices includes indirect taxation, $T_{t}^{d}$ , on top of payments to labor and capital. Moreover, the mixed income payments from the household sector, ${\text{MI}}_{t}^{d}\left(\text{HH}\right)$ , include a large proportion of payments to labor services provided by the business owner and her family (see CKR for details). Due to these two issues, CKR argue that a more appropriate measure of the empirical labor share is

(21) $$1-\alpha_{t}^{d}=\frac{{\text{CE}}_{t}^{d}-{\text{CE}}_{t}^{d}\left(\text{HH}\right)}{Y_{t}^{d,n}-{\text{CE}}_{t}^{d}\left(\text{HH}\right)-MI_{t}^{d}\left(\text{HH}\right)-T_{t}^{d}},$$

where ${\text{CE}}_{t}^{d}\left(\text{HH}\right)$ is compensation of employees from the household sector.

We repeat the analysis from the benchmark using the CKR labor share. We report the results from the analysis in Table 5. We find that the use of an alternative way to compute the labor share does not affect our main results: no model is dominant and the relative errors across models are very similar. In particular, *Constant* still performs best in two out of seven series (capital and labor) and comes in second in three more (interest rate, consumption, and output per worker). Interestingly, in this case it is *Trend* that dominates in more variables (interest rate, wage, investment and capital), whereas *Exact* is best at predicting output per worker.

**Table 5:** Relative errors, CKR labor share.

	*r*	*w*	*I*	*C*	*K*	*L*	*Y*/*L*
Constant	0.1155	0.0490	0.2545	0.1313	**0.2107**	**0.1569**	0.0310
Trend	**0.1008**	**0.0421**	**0.2314**	**0.1296**	0.2355	0.1647	0.0389
Exact	0.1192	0.0450	0.2532	0.1327	0.2134	0.1570	**0.0293**

### 4.3 CES Production Function and Falling Price of Investment

**Figure A.1:**
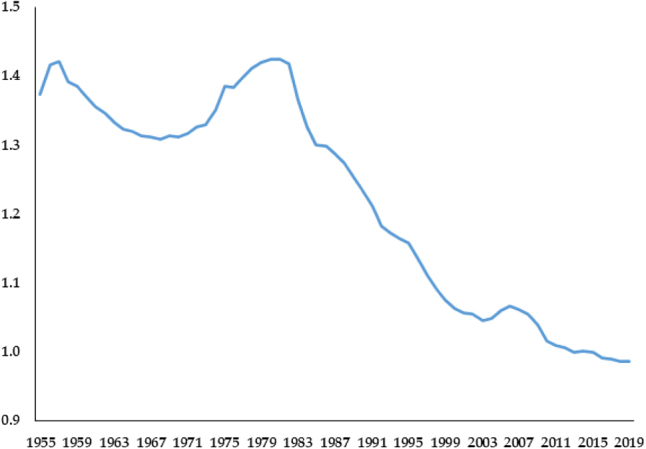
Falling price of investment. Normalized to one in 2010.

We last consider an alternative production function to Cobb–Douglas. It is well-known that the period we study coincides with a secular decline in the relative price of investment. The time series of a falling price of investment that we obtain can be found in Appendix A, Figure A.1. Karabarbounis and Neiman (2014) show that this fall in relative price, together with a CES production function, can generate half of the decline in the labor share observed in the data. Here we compare our benchmark results to using a CES production function.

The model in Section 2 has only one sector, implying that the relative price of investment is constant and equal to one. To overcome this, we build a (well-known) two-sector growth model, where household’s investment, *I*_*t*_, is transformed into new capital. This transformation is done by the investment sector, whose productivity *Z*_*I*,*t*_ can change over time. Compared to the NGM presented in Section 2, Eq. (2) becomes

(22) $$C_{t}+I_{t}\leq w_{t}L_{t}+r_{t}K_{t},$$

where

(23) $$K_{t+1}-\left(1-\delta \right)K_{t}=Z_{I,t}I_{t}.$$

With *Z*_*I*,*t*_ growing over time, the relative price of investment becomes cheaper.

A CES production function implies that output is given by

$$Y_{t}={\left(\alpha K_{t}^{\frac{\sigma -1}{\sigma }}+\left(1-\alpha \right){\left(E_{t}L_{t}\right)}^{\frac{\sigma -1}{\sigma }}\right)}^{\frac{\sigma }{\sigma -1}},$$

where *α* is the weight of capital in final output, *σ* is the elasticity of substitution between capital and labor (following KN, we set it to 1.25), and *E*_*t*_ is the labor-augmenting productivity. In this model, the labor share is given by

(24) $$\frac{w_{t}L_{t}}{Y_{t}}=\left(1-\alpha \right)\frac{{\left(E_{t}L_{t}\right)}^{\frac{\sigma -1}{\sigma }}}{\alpha K_{t}^{\frac{\sigma -1}{\sigma }}+\left(1-\alpha \right){\left(E_{t}L_{t}\right)}^{\frac{\sigma -1}{\sigma }}}.$$

Note that the labor share is not constant: the falling price of investment makes *K*_*t*_ grow relatively faster than *E*_*t*_ *L*_*t*_, implying a falling labor share. In this exercise we calibrate a new series for *α* ^CES^ so that the average of the right hand side in Eq. (24) is equal to the average in the left hand side.

As before, we simulate seven macro aggregates for the CES model, and compare it to the benchmark values of the model *Constant* in Table 6. The CES model is outperformed in six of the seven variables. In fact, both *Trend* and *Exact* also outperform the CES model in those six macro-aggregates (see Table 1). The only variable where the CES dominates is in capturing well the amount of hours worked. This result is important to highlight that, again, more richness in the model (CES structure, falling prices of investment) does not necessarily imply improved accuracy when predicting macro aggregates.

**Table 6:** Relative errors, CES production function and falling price of investment.

	*r*	*w*	*I*	*C*	*K*	*L*	*Y*/*L*
CES	0.2285	0.1240	1.0394	0.2103	0.5107	**0.1522**	0.1522
Constant	**0.1631**	**0.1037**	**0.5378**	**0.1016**	**0.4335**	0.1834	**0.0874**

The quantitative results from this section tell us that the labor share decline, while stark compared to Kaldor’s stylized fact, has little impact on the performance of a calibrated NGM. This message is also robust, surviving alternative time periods, computation of the labor share, and accounting explicitly for the declining price of investment in a two-sector model.

In the next section we turn to the question of why performance across models is so similar. After all, the labor share does decline by more than six percentage points between 1970 and 2010. We illustrate how mechanically reducing the labor share would yield paths of wages and interest rates closer to the data, but general equilibrium responses undo this.

## 5 Stagnant Wages and the Labor Share

In this section we investigate the importance of general equilibrium responses to a labor share decline. Our focus on wage series in the model is driven by concern in recent years over the causes and implications of sluggish wage growth (see e.g. Stansbury and Summers (2017) and Kehrig and Vincent (2020)). The labor share is the fraction of output accruing to workers which, divided by hours, is the wage. It would seem natural then that labor’s declining share of output should translate into lower wage growth. In this section we show that this connection falls apart when taking into account general equilibrium effects. Our counterfactual exercise demonstrates the strength of these general equilibrium effects, while also highlighting the difficulty that theories of the labor share decline may have in matching empirical wage movements.

Recall that our empirical series for wages, Eq. (9), is given by the product of the labor share and labor productivity. In a balanced growth path, the first term is constant and the second term grows at a constant rate. The mechanical impact of a falling labor share is to reduce the first term, slowing wage growth. We construct a naive, counterfactual wage series following this logic. We compute this as

(25) $$w_{t}^{n}=\left(1-\alpha_{t}^{d}\right)\frac{Y_{t}^{c}}{L_{t}^{c}},$$

where *n* stands for naive, we use the empirical labor share for the first term, and the predicted paths of output and hours worked by the NGM with a constant labor share for the second term.

**Figure 8:**
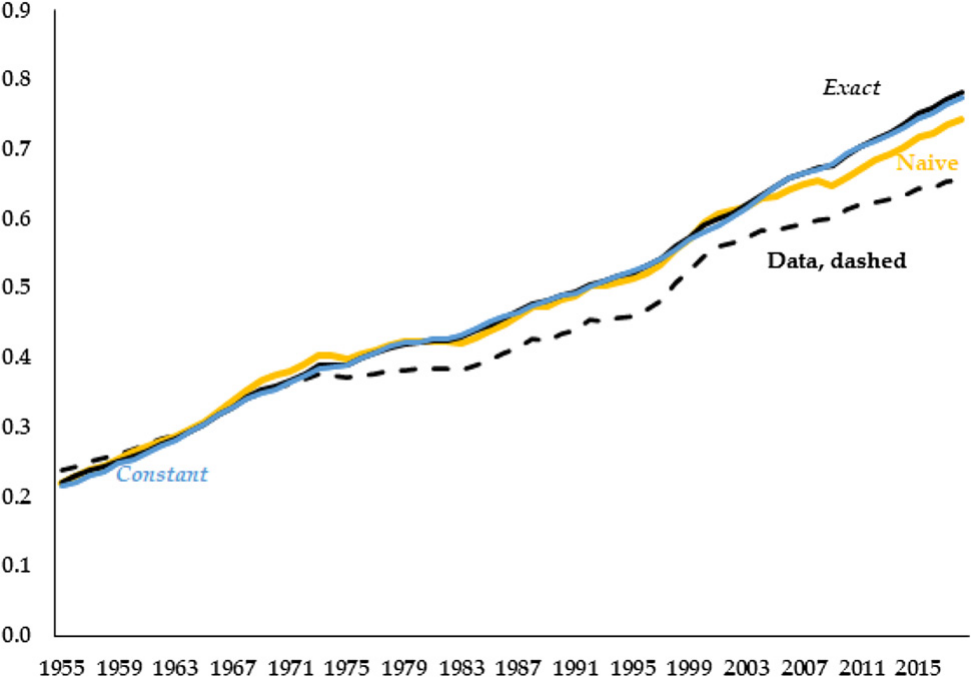
Wage series. Data, constant, and naive.

In Figure 8 we plot, in yellow, the naive series from Eq. (25) against the black, dashed line representing our empirical wage series from Eq. (9). The naive series does a better job than *Constant*, particularly in capturing the observed slowdown in wage growth from the late 1990s. The wage series predicted by the *Constant* model, in blue, grows continuously, picking up none of the sluggish wage growth in the latter part of the sample. In fact, the relative error of the naive series is 0.0874, compared to the 0.1037 from *Constant* (by comparison, this error is also larger, at 0.1071 in *Trend*, and 0.1035 at *Exact*).

**Figure A.2:**
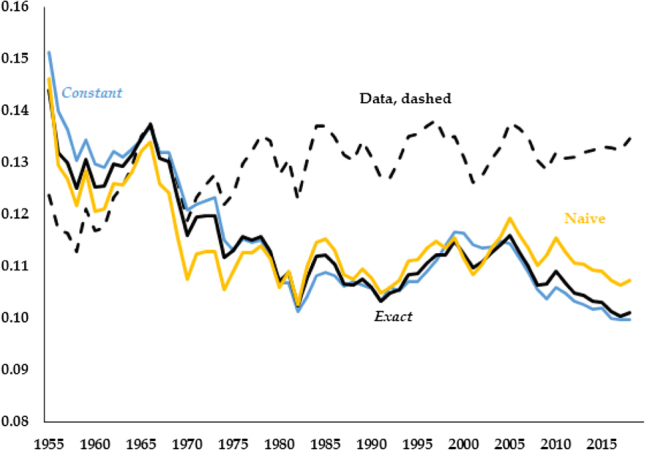
Interest rate. Data, constant, and naive.

To some degree our naive counterfactual does appear to indicate a connection between the labor share and wage stagnation – the timing of the labor share decline seems consistent with the slow down in wage growth. However, our naive measure excludes all endogenous responses to a falling labor share. As noted earlier, a labor share decline raises the return to capital, fostering an increase in investment and the capital stock. This raises the marginal product of labor and wages. Quantitatively, we find that this second effect is about as large as the mechanical effect initially highlighted. The black, solid line in Figure 8, which is the equilibrium wage path from the *Exact* model, illustrates this. In spite of the decline in the labor share, the predicted wage series lies on top of its counterpart from a model where the labor share is constant (note that the black line can barely be seen under the blue one). A similar exercise for interest rates demonstrates the same equilibrium forces for that variable. The resulting graph is presented in Appendix A, Figure A.2.

In empirical work Stansbury and Summers (2017) recently conclude that factors other than productivity are responsible for wage stagnation. Kehrig and Vincent (2020) analyze the aggregate labor share decline and find that the lack of wage growth is driven by reallocation of value added to establishments with a low and declining labor share. Through the lens of an NGM however, our exercise suggests that a fall in the labor share cannot alone generate the observed wage stagnation. General equilibrium effects completely offset the mechanical decrease in wage growth. More broadly, the similarity of model performance in Table 1 is not the result of small changes to the labor share but rather strong dynamic general equilibrium responses that counter the labor share decline.

## 6 Final Remarks

The evolution of the labor share has received widespread attention in recent years. In this paper we assess the quantitative importance of a changing labor share through the lens of a benchmark macroeconomic model. Simulating three versions of the NGM, we find that accounting for this evolution has little impact on model performance. Further, we show that the similar performance across models is due to strong general equilibrium effects. For example, a counterfactual exercise ignoring equilibrium responses to the labor share decline yields a markedly different path of wages, and actually generates wage stagnation consistent with the data. Allowing for equilibrium responses entirely undoes this, yielding a path of wages nearly identical to the model with a constant labor share.

As a whole, our analysis finds that for a standard set of macro aggregates the NGM is still an appropriate choice. We argue that these results give needed quantitative context to the debate around the labor share decline and its importance in macro models. This is especially true given the large number of macro models with the NGM at their foundation.
